# AXL modulates extracellular matrix protein expression and is essential for invasion and metastasis in endometrial cancer

**DOI:** 10.18632/oncotarget.12637

**Published:** 2016-10-13

**Authors:** Laura M. Divine, Mai R. Nguyen, Eric Meller, Riva A. Desai, Batool Arif, Erinn B. Rankin, Katherine H. Bligard, Cherise Meyerson, Ian S. Hagemann, Maria Massad, Premal H. Thaker, Andrea R. Hagemann, Carolyn K. McCourt, Matt A. Powell, David G. Mutch, Katherine C. Fuh

**Affiliations:** ^1^ Department of Obstetrics and Gynecology, Washington University School of Medicine, St. Louis, MO, USA; ^2^ Division of Radiation and Cancer Biology, Department of Radiation Oncology, Stanford University Medical Center, Stanford, CA, USA; ^3^ Department of Obstetrics and Gynecology, Stanford University Medical Center, Stanford, CA, USA; ^4^ Department of Pathology and Immunology, Washington University School of Medicine, St. Louis, MO, USA; ^5^ Center for Reproductive Health Sciences (CRepHS), Washington University in St. Louis, St. Louis, MO, USA

**Keywords:** AXL, endometrial cancer, uPA, MMP, ARK1

## Abstract

The receptor tyrosine kinase AXL promotes migration, invasion, and metastasis. Here, we evaluated the role of AXL in endometrial cancer. High immunohistochemical expression of AXL was found in 76% (63/83) of advanced-stage, and 77% (82/107) of high-grade specimens and correlated with worse survival in uterine serous cancer patients. *In vitro*, genetic silencing of AXL inhibited migration and invasion but had no effect on proliferation of ARK1 endometrial cancer cells. AXL-deficient cells showed significantly decreased expression of phospho-AKT as well as uPA, MMP-1, MMP-2, MMP-3, and MMP-9. In a xenograft model of human uterine serous carcinoma with AXL-deficient ARK1 cells, there was significantly less tumor burden than xenografts with control ARK1 cells. Together, these findings underscore the therapeutic potentials of AXL as a candidate target for treatment of metastatic endometrial cancer.

## INTRODUCTION

Endometrial cancer (EC) is the most common gynecologic malignancy in the United States with 60,050 new cases and 10,470 disease-related deaths projected for 2016. The prognosis for early-stage cancer has a five-year survival rate of 80% to 95% [1, 2]. However, about 29% of patients are diagnosed with aggressive tumors that have spread to regional lymph nodes or metastasized to more distant areas [1]. In patients with distant EC, the five-year survival rate is only 17% [1]. The more aggressive variant of type II EC, uterine serous cancer, accounts for only 10% of all EC cases but is responsible for 40% of deaths by this disease [3, 4]. Currently, the standard treatment for EC consists of surgery followed by hormonal therapy, chemotherapy, and/or radiation therapy, depending on patient age and comorbidities, tumor stage and grade, performance status, and prior treatments [5]. Although chemotherapy is the treatment of choice for metastatic or recurrent EC, median survival is only about 12 months for these patients [6-8]. High mortality and limited treatment options underscore the challenge of metastatic disease. Developing effective treatments will require a better understanding of EC metastasis and identification of more effective therapeutic targets.

The receptor tyrosine kinase AXL has been identified as a novel therapeutic target given its involvement in tumor invasion and migration. As a member of the TAM family of receptor tyrosine kinases, AXL contains an extracellular domain composed of two immunoglobulin-like domains and two fibronectin type III domains [9, 10]. The extracellular domain resembles that of adhesion molecules, and overexpression of AXL causes an adhesive phenotype [11, 12]. Binding of growth arrest specific gene-6 (GAS-6), the only known ligand for AXL, induces autophosphorylation of tyrosine residues 779, 821, and 866 [13-15]. Activation of AXL stimulates several signal transduction pathways, including MAPK/ERK kinases, PI3K/AKT, NF-κB, and STAT and promotes proliferation, anti-apoptosis, survival, cellular migration, invasion, adhesion, and pro-inflammatory cytokine production [14, 16-21].

AXL is overexpressed or ectopically expressed in multiple cancers including breast, prostate, ovarian, and endometrial cancer [10, 22-29]. In tumor cells, AXL over-expression promotes migration and invasion, whereas AXL inhibition decreases cell invasion and increases chemosensitivity [29-31]. *In vivo*, AXL signaling promotes metastasis in breast, glioma, lung, and ovarian cancer [24, 32-36]. These findings demonstrate that AXL is an important driver of tumor metastasis.

AXL overexpression has been reported in human uterine cancer [27], but the role of AXL in EC progression has yet to be elucidated. Using both *in vitro* and *in vivo* approaches, we demonstrate that AXL is critical in EC invasion, migration, and metastasis. Thus, our study supports the therapeutic potential of targeting AXL to inhibit metastatic EC.

## RESULTS

### AXL is upregulated in endometrial cancer tissues

**Figure 1:**
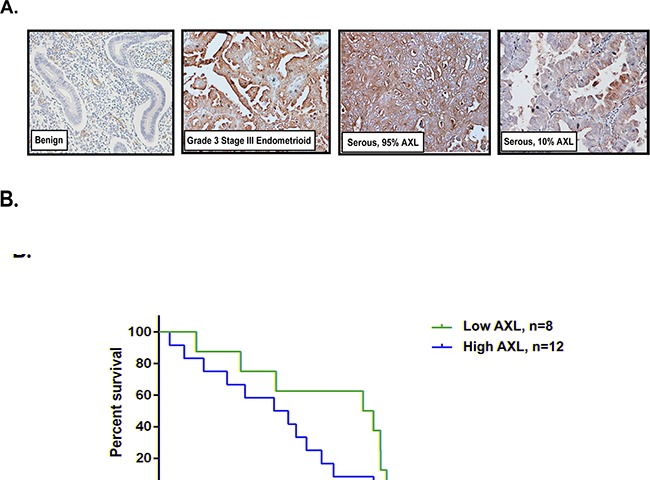
High AXL expression in endometrial tumors correlates with lower survival **A.** Representative images of AXL immunohistochemical staining in benign endometrial glands, high grade endometrioid, and serous carcinomas at 20X magnification. **B.** Kaplan-Meier curves showing differences in survival between low AXL and high AXL expressing uterine serous tumors. Tumors expressing low AXL (5-10%, n=8) have survival median of 96 months while tumors expressing high AXL (60-100%, n=12) have significantly lowered survival of 56 months (P=0.0285).

To confirm and extend previous reports of AXL overexpression in human EC samples, we performed immunohistochemical analysis of human endometrial tissue microarrays that included benign endometrial glands, endometrioid cancer, serous adenocarcinoma, and several other mixed cancer types and metastases (Table 1 and Figure 1A). Tissues were scored on a 0–3 scale by the percentage of AXL-positive cells. Our data show that whereas 24% (15/63) of normal specimens expressed high levels of AXL (score 2, or 3), 63% (180/287) of primary type I and type II endometrial carcinoma samples and 86% (286/332) of endometrial cancer in all stages (primary tumors and metastases) expressed AXL (*P*<0.0001 for both, Table 1). Therefore, AXL was more highly expressed in malignant endometrium than in normal endometrium. Additionally, tumor samples collected from metastatic sites showed significantly higher expression of AXL than normal samples; whereas 85% (11/13) of metastatic carcinoma samples expressed AXL and 46% (6/13) received a score of 3, only 43% (27/63) of normal samples expressed AXL and only 5% (3/63) received a score of 3 (*P*=0.0001, Table 1). Advanced-stage and high-grade endometrial carcinomas also expressed more AXL than early-stage and low-grade carcinomas. For example, 19% of stage I carcinomas received an AXL score of 3, versus 51% of stage III and 38% of stage IV carcinomas (P=0.0003, Table 1). Similarly, only 14% of grade 1 carcinomas were scored as 3 versus 45% of grade 3 carcinomas (P<0.0001, Table 1). These results indicate that AXL expression was significantly elevated in primary endometrial cancer tumors, especially in higher-grade and advanced stage tumors and metastatic sites.

**Table 1:** AXL expression in human endometrial cancer Immunohistochemical analysis of AXL staining in normal endometrial glands, human endometrial tumors and metastases

Analysis of AXL Staining to Endometrial Tumor Parameters					
Score	0	1	2	3	Total
Benign/Atrophic Endometrium	36 (57)	12 (19)	12 (19)	3 (5)	63
Type I					
Endometrioid	46 (18)	51 (19)	67 (26)	96 (37)	260
Type II					
Serous	2 (8)	5 (21)	6 (25)	11 (46)	24
Clear-Cell	2 (67)	1 (33)	0	0	3
Mixed	1 (5)	5 (23)	8 (36)	8 (36)	22
Stromal Sarcoma	1 (25)	3 (75)	0	0	4
Carcinosarcoma	0	2 (25)	2 (25)	4 (50)	8
Metastatic Carcinoma	2 (15)	3(23)	2(15)	6(46)	13
Stage^a^					
I	35 (16)	37 (18)	99 (47)	41 (19)	212
II	5 (14)	7 (19)	12 (32)	13 (35)	37
III	6 (8)	10 (13)	21 (28)	38 (51)	75
IV	2 (25)	2 (25)	1 (12)	3 (38)	8
Grade					
1	20 (35)	10 (17)	20 (34)	8 (14)	58
2	12 (12)	16 (16)	39 (38)	34 (34)	101
3	10 (9)	15 (14)	34 (32)	48 (45)	107

Values are presented as n (%). AXL staining score: 0, 0% AXL-expressing cells; 1, 1%–19%; 2, 20%–59%; 3, >60%.

a Of the 334 total specimens, stage was available for 332 of these specimens.

In a subset of 20 patients for whom we had clinico-pathological and survival information, 8 patients with low AXL expression had longer median survival (96 months) than the 12 patients with high AXL expression (96 vs. 56 months, *P*= 0.0285, Figure 1B).

### AXL promotes invasion and migration of endometrial cancer cells

**Figure 2:**
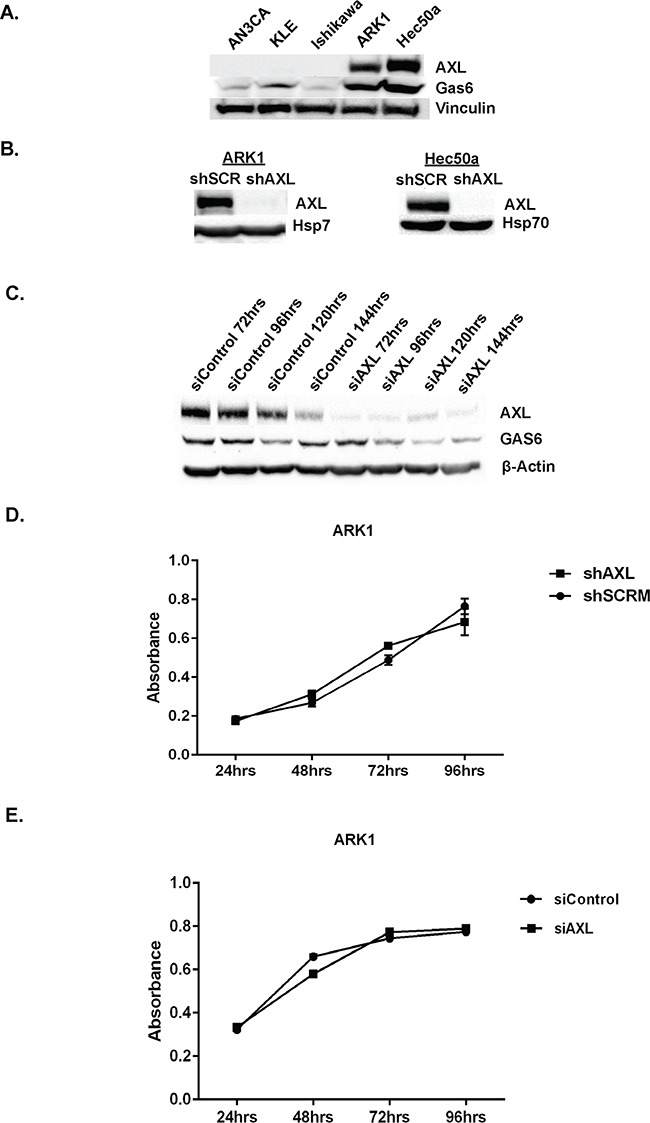
Genetic inactivation of AXL does not affect proliferation of endometrial cancer cell line **A.** Western blot analysis of AXL (140 kDa) and GAS6 (75kDa) expression in a panel of human endometrial cancer cell lines. Vinculin (125 kDA) is shown as a loading control. **B.** Western blot analysis of AXL (140 kDa) expression in ARK1 and Hec50a cells stably transfected with shRNA targeting AXL (shAXL) or scramble control (shSCRM). Heat shock protein 70 (Hsp70, 70 kDa) is shown as a protein loading control. **C.** Western blot analysis of AXL (140 kDa) and GAS6 (75kDa) expression in siControl and siAXL ARK1 cells at 72, 96, 120, and 144 hours post transfection. β-actin (42 kDa) is shown as a loading control. **D.** Cellular growth curves for ARK1 shSCRM and shAXL cells (n=3). Data are represented as mean +/− SEM. **E.** Cellular growth curves for siControl and siAXL ARK1 cells (n=3). Cells were plated into 96 wells plate for XTT proliferation assay 48hrs after siRNA transfection. Data are represented as mean +/− SEM.

To define the role of AXL in endometrial cancer, we first screened five EC cell lines for AXL expression. AXL was abundantly expressed in two cell lines derived from type II metastatic EC, ARK1 and Hec50a (Figure 2A). In contrast, AXL was expressed at very low levels in AN3CA (derived from a grade 3 endometrioid adenocarcinoma), KLE (derived from a poorly differentiated adenocarcinoma), and Ishikawa (derived from a well differentiated type I adenocarcinoma) cells (Figure 2A).

To examine the role of AXL in proliferation of EC cells, we used short hairpin RNAs (shRNAs) to generate AXL-deficient metastatic endometrial cancer cell lines (ARK 1 and Hec50a) as described previously [37] and confirmed AXL knockdown by Western analyses (Figure 2B). We then used an XTT-based assay to measure proliferation of ARK1 cells containing scrambled shRNA (shSCRM) or AXL-targeting shRNA (shAXL). Over a period of 4 days, we found no significant difference in cellular growth curves (Figure 2D). Consistent with this finding, we examined ARK1 cells transfected with small interfering RNA (siAXL or siControl, Figure 2C) and found no difference in growth curves (Figure 2E). Thus, we conclude that AXL is not necessary for endometrial cancer cell proliferation *in vitro*.

**Figure 3:**
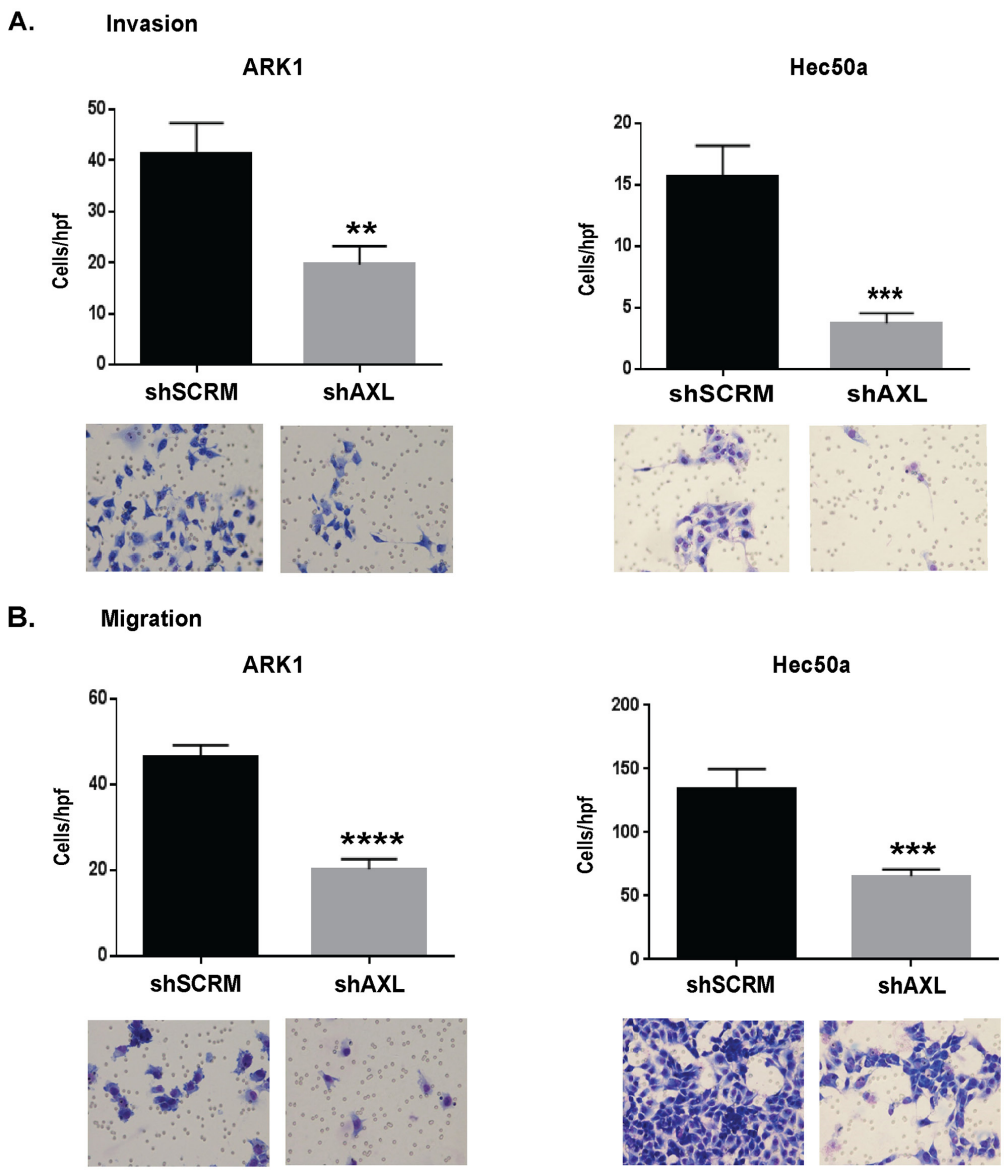
AXL is essential to invasion and migration of endometrial cancer cells **A.** Matrigel invasion assay of ARK1 and Hec50a shSCRM and shAXL cells. Images were taken after 48h at 20X magnification showing fewer invading cells for AXL deficient cells. Graphs show quantification of invasion assays (n=3). Data are represented as mean +/− SEM and asterisks indicate a significant increase or decrease in expression compared with shSCRM as determined by student's t test (**, P < 0.01; ***, P < 0.001). **B.** Migration assay using boyden chambers of shSCRM and shAXL ARK1 and Hec50a cells. Images were taken after 48h at 20X magnification illustrating inhibition of shAXL cell migration. Graphs show quantification of migration assays (n=3). Data are represented as mean +/− SEM and asterisks indicating statistical significance compared to shSCRM as determined by the student's t-test (***, P < 0.001; ****, P <0.0001).

To evaluate the contribution of AXL to tumor cell invasion, we performed matrigel invasion assays and found that AXL-deficient ARK 1 and Hec50a cells were significantly less invasive than controls (Figure 3A). ARK1 cells transfected with AXL siRNA were similarly inhibited in their invasive ability (Supplementary Figure S1A). Likewise, shAXL ARK1 and Hec50a and siAXL ARK1 cells were less able to migrate in a transwell assay than the respective control cells (Figure 3B and Supplementary Figure S1B).

**Figure 4:**
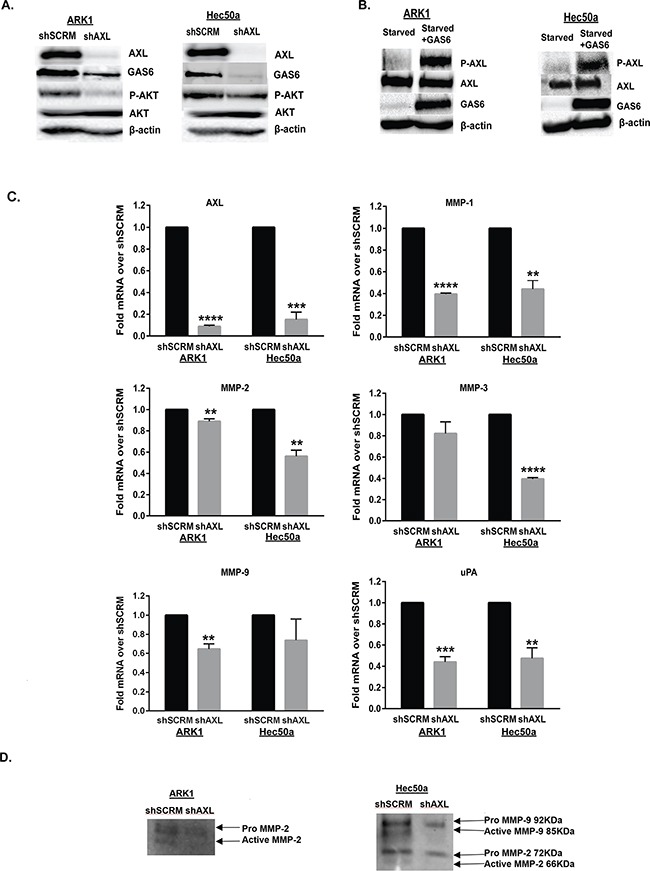
AXL regulates PI3K/AKT signaling pathway and MMP expression in endometrial cancer cells **A.** Western blot analysis of phospho-AKT (Ser473, 56-60 kDa), total AKT (60 kDa), AXL (140 kDa), and GAS6 (75 kDa) expression in ARK1 and Hec50a cells expressing shSCRM or shAXL. β-actin (42 kDa) is shown as a loading control. **B.** Western blot analysis of AXL (140 kDa), phospho-AXL (Tyr702, 140 kDa) and GAS6 (75 kDa) expression in starved ARK1 and Hec50a cells with or without GAS6 stimulation. β-actin (42 kDa) is shown as a loading control. **C.** Real time PCR analysis of MMP-1,-2,-3,-9, uPA, and AXL expression in shAXL and shSCRM endometrial cancer ARK1 and Hec50a cells. Expression values were normalized to 18S and fold changes were calculated over shSCRM (n=3). Data are represented as mean +/− SEM and asterisks indicate significant increase or decrease in expression compared to shSCRM as determined by the student's t-test (**, P < 0.01; ***, P < 0.001; ****, P <0.0001). **D.** Gelatin zymography assay for pro- and active-MMP2 and MMP-9 activity in conditioned media collected from serum starved Hec50a cells.

We further examined the effects of therapeutic inhibition of AXL using a small molecule inhibitor R428[34]. We first confirmed the effectiveness of R428 in inhibiting p-AXL expression after stimulation with GAS6 (Supplementary Figure S2A). We then found that pre-treatment of ARK1 with R428 for four hours inhibited invasion (Supplementary Figure S2B). Thus, our data shows that AXL plays an important role in invasion and migration of uterine serous cancer cells and that its inhibition would significantly hinder these processes. We next sought to determine the molecular mechanism by which AXL regulates EC cell invasion and migration. We were particularly interested in the PI3K/AKT pathway because AXL has been reported to regulate ovarian cancer metastasis through this pathway [24]. To determine whether PI3K/AKT signaling is affected by loss of AXL in endometrial cancer cells, we performed Western blot analyses evaluating levels of AKT phosphorylated at Ser473 (P-AKT) on shSCRM and shAXL ARK1 and Hec50a cells. Protein expression on western blot confirmed that AXL-deficient ARK1 and Hec50a cells have significantly reduced P-AKT and GAS6 expression than the shSCRM counterpart (Figure 4A).

To determine whether AXL activation in uterine cancer is ligand dependent, we starved ARK1 and Hec50a cells for 48 hours. Then exogenous GAS6 was added at 600ng/ml concentration for 15mins to stimulate AXL activation. Western blot analysis showed that exogenous GAS6 induced phosphorylation of AXL in both endometrial cancer cell lines (Figure 4B).

To examine components downstream of PI3K/AKT pathway, we focused on matrix metalloproteinases (MMPs) because they have been found to be major contributors to tumor cell invasion and metastasis [38-40]. Expression of MMP-1 and MMP-2 has been reported to be associated with EC cell invasion [41, 42]. Additionally, AXL has been shown to regulate MMP-1, MMP-2 and MMP-9 expression in ovarian cancer [24]. Likewise, MMP-3 has been associated with vascular invasion in EC and AXL has been reported to induce MMP-3 expression in head and neck cancer cell invasion [43, 44]. We also examined expression of urokinase-type plasminogen activator (uPA) since it has also been reported to enhance EC cell invasion via phosphorylation of AKT [45]. We found that AXL-deficient ARK1 cells expressed significantly less MMP-1, MMP-2, MMP-9, and uPA than controls (Figure 4C). Similarly, AXL-deficient Hec50a cells expressed significantly less MMP-1, MMP-2, MMP-3, and uPA than control cells (Figure 4C). To assess functional measures, gelatin zymogram for MMP-2 and MMP-9 was performed on Hec50a and ARK1 shSCRM and shAXL cells. Hec50a shAXL showed significantly less active MMP-2 and MMP-9, and ARK1 shAXL had less active MMP-2 than shSCRM cells (Figure 4D). Taken together, our results suggest that AXL regulates MMPs and uPA expression through the PI3K/AKT pathway in EC cells.

### Genetic inhibition of AXL significantly reduces endometrial cancer metastasis in mice

**Figure 5:**
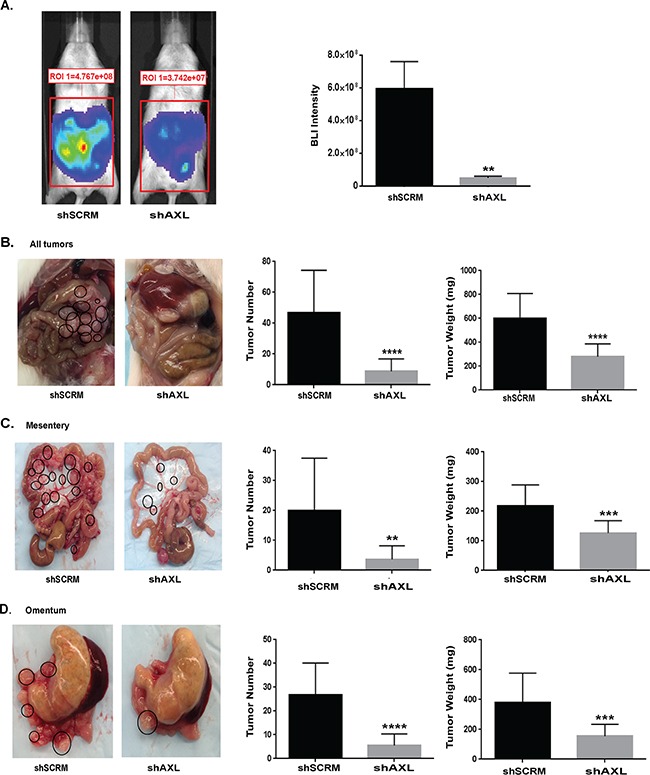
Genetic inactivation of AXL significantly inhibits endometrial metastasis in mouse model **A.** Bioluminescent imaging (BLI) of mice injected with luciferase-tagged ARK1 shSCRM and shAXL cells at 35 days following injection. Graph on the right shows quantification of BLI intensity. Data are represented as mean +/− SEM and asterisks indicating statistical significance as determined by the student's t-test (**, P < 0.01). **B.** Representative images of abdominal cavity taken ~60 days after injection with shSCRM and shAXL ARK1 cells. Note that mice injected with shSCRM cells visually displayed more tumor metastasis throughout the abdominal cavity than shAXL mice (circled). Graphs depict the average number of all peritoneal metastases >1mm in size per mouse and the average weight of total tumor nodules from each treatment group (n=9 per group). Data are represented as mean +/− SEM and asterisks depict significant change in tumor burden as determined by student's t test (****, P < 0.0001). **C.** Images of the mesentery from mice injected with shSCRM or shAXL ARK1 cells. Note that tumor nodules are circled. Graphs on the right depict average number of tumor nodules and tumor weight from the mesentery alone. Data are represented as mean +/− SEM and asterisks depict significant change in tumor burden as determined by student's t test (**, P < 0.01; ***, P < 0.001). **D.** Images of the omentum from mice injected with shSCRM or shAXL ARK1 cells. Graphs on the right depict average number of tumor nodules and tumor weight from the omentum alone. Data are represented as mean +/− SEM and asterisks depict significant change in tumor burden as determined by student's t test (***, P < 0.001; ****, P < 0.0001).

Finally, we sought to examine the role of AXL in EC metastasis *in vivo*. Since advanced-stage EC presents with intraperitoneal metastasis, we used a mouse model in which NOD-SCID mice are intraperitoneally injected with ARK1 cells. In this model, mice develop ascites and many small metastatic lesions attached to the mesentery, omentum, and other peritoneal surfaces [46]. We intraperitoneally injected mice with luciferase labeled ARK1 cells either shAXL or shSCRM. After 35 days, we performed in vivo evaluation using bioluminescence imaging (BLI) to ensure tumor growth and metastases development in the injected mice. BLI quantification highlighted the difference in the ability of shSCRM and shAXL ARK 1 to form metastases. Specifically, ARK1 shSCRM had significantly higher mean BLI intensity (5.9×10^8^±1.7×10^8^) than those injected with ARK1 shAXL cells (5.1×10^7^±1.1×10^7^), indicating higher tumor burden (P=0.0085, Figure 5A). After 60 days, we counted and weighed the tumors. Mice injected with AXL-deficient cells developed fewer intraperitoneal metastases and had lower tumor weight than mice injected with AXL-expressing cells (Figure 5B, 5C and 5D). The average number of peritoneal metastases was significantly reduced from 47±8 in shSCRM injected mice to 9 ±2 in shAXL injected mice (P<0.0001, Figure 5B). Likewise, the average tumor weight was reduced greatly from 598 ± 63mg in shSCRM injected mice to 281 ± 29mg in shAXL injected mice (Figure 5B, P< 0.0001). We observed similar reductions in tumor number and weight when the tumors were divided according to the site of metastasis, either in the mesentery or omentum (Figure 5C and 5D). Overall, these findings demonstrate that knockdown of AXL expression in ARK 1 cells inhibited endometrial cancer metastasis. This is the first *in vivo* experiment to establish the essential role of AXL in the development of EC metastasis.

We further evaluated whether tumors seen in the shAXL model expressed AXL, and we found that a portion of tumors that grew either had AXL protein expression or a lack of AXL expression. Therefore tumor growth was due to either incomplete knockdown or growth despite knockdown (Supplementary Figure S3A). At the mRNA level, AXL knockdown tumors were found to have low expression of AXL (Supplementary Figure S3B). In addition, shAXL tumors had less p-AKT protein expression, uPA, MMP-1, MMP-2, MMP-3 and MMP-9 mRNA expression as seen in vitro. To further confirm whether AXL regulates metastasis rather than proliferation, Ki67 expression was evaluated in the xenograft tumors and shAXL tumors had similar Ki67 expression as shSCRM (Supplementary Figure S4). This is consistent with our findings that AXL inhibition decreases tumor growth via inhibition of metastasis rather than by proliferation.

## DISCUSSION

Metastatic and recurrent EC pose challenges to current therapies as median patient survival is less than one year [6-8]. Moreover, current chemotherapies for advanced EC can result in significant morbidity, leading to poor tolerability and ultimately discontinuation in some cases [47]. However, advances in the understanding of the molecular mechanisms underlying cancer have made it possible to use targeted agents to disrupt cancer cell progression and survival. This targeted approach may be able to reduce systemic side effects and improve quality of life and overall survival for patients [47, 48].

To date, there are few receptor tyrosine kinases that have been identified as major factors in EC invasion. More specifically, the biological role of AXL, a receptor tyrosine kinase, in EC remains largely uncharacterized despite its promising advances in numerous cancers. In this study, we confirmed that AXL was significantly upregulated in EC tissues and correlated with advanced stage and worse survival. Our sample size for the survival analysis was limited to 8 patients for the low expression AXL group and 12 for the high AXL expression group, and other confounding factors such as age, chemoresistance, and other pathology risk factors were unavailable for analysis. Future studies are needed to confirm these findings. Nonetheless, this is the first data to implicate AXL as a clinically relevant prognostic factor in EC, in particular, associated with worse prognosis.

Furthermore, we demonstrated that genetic knockdown of AXL significantly inhibited EC invasion and metastasis both *in vitro* and *in vivo*. Pharmacological inhibition using R428 was also used to inhibit endometrial cancer cell invasion in vitro, suggesting clinical relevance of AXL inhibition. As in ovarian cancer, reduction of AXL expression primarily reduced the establishment of new metastatic lesions during progression of EC metastasis rather than reducing proliferation of the established tumors themselves [24]. Immunohistochemical analysis of Ki-67 expression in ARK1 shAXL and shSCRM mouse xenografts confirms that AXL knockdown has no significant effect on Ki-67. This is consistent with our proliferation data in which AXL displayed no effect on the proliferation and growth of ARK1 cells. AXL has been shown to regulate proliferation and survival in a number of cancers; however, it appears that AXL signaling and overall biological effect differs depending on tumor type. This study provides more support that AXL may inhibit invasion but not proliferation as seen in ovarian cancer [24]. Although the majority of anti-cancer agents are directed at growth inhibition, most cancer patients are affected by metastasis [49]. Few drugs have been proven effective at treating cancer metastasis [49]. Our data suggests AXL to be a promising candidate for inhibiting EC metastasis.

We have demonstrated ligand-dependent activation of AXL in EC. Starved ARK1 cells had low levels of endogenous GAS6 and minimal p-AXL. Upon stimulation with exogenous GAS6, AXL phosphorylation was induced. Additionally, GAS6 levels correlated with AXL expression in EC cell lines as well as a decreased level in cells with genetic inactivation of AXL. Thus indicating GAS6 to be an important factor to consider with AXL inhibition. We also showed that AXL regulation of EC is likely through the PI3K/AKT pathway. Given the importance of the PI3K/AKT pathway in gynecologic carcinogenesis, our findings suggest that AXL might be a novel target to inhibit upstream of this pathway. Moreover, we demonstrated that silencing AXL significantly inhibited the expression of proteins involved in the regulation of the extracellular matrix such as MMPs and uPA, unveiling the pathway by which AXL mediates tumor metastasis. Urokinase-type plasminogen activator (uPA), an important player in the degradation of the extracellular matrix, is associated with worse prognosis in EC [45, 50]. Furthermore, uPA has been reported to boost EC cell invasion via increased levels of p-AKT, p-ERK1/2 and p-p38 [45]. This is the first time that uPA has been shown to be regulated by AXL, possibly through the p-AKT pathway. This finding illuminates a more complete mechanism by which AXL and its downstream proteins interact to enhance cancer invasion.

Overall, current molecular-targeted agents against EC exhibited low impact on survival and response in clinical trials, suggesting that further studies are needed to address the problems with current agents and identify more effective molecular targets [47]. Taken together, our data suggest that AXL may be a novel therapeutic target to inhibit endometrial cancer metastasis. In fact, AXL-specific inhibitors and multi-kinase inhibitors targeting AXL are already under development for treatment of several cancers [10]. For example, SKI606 (Bosutinib), a multi-kinase inhibitor targeting AXL, has been shown to reduce AXL-specific invasiveness in hepatocellular carcinoma cells and is in Phase 1 and 2 clinical trials for metastatic breast cancer [10, 51]. Additionally, R428 (BGB324), a small molecule inhibitor of AXL, used in our *in vitro* experiments, is currently in clinical trials in acute myeloid leukemia and in non-small cell lung cancer. AXL inhibition via soluble AXL receptors *in vivo* has also been shown to reduce ovarian cancer metastasis in mice with established disease by 63% [24].

There has yet to be any study to target AXL in advanced EC. Thus, our findings may serve as a foundation for more studies on AXL in EC since AXL expression positively correlates with mesenchymal markers, increased metastasis, and drug resistance in various solid tumors [10, 32, 52-57]. Thus, these results may enable targeting AXL not only for invasion, but also for drug resistance and other associated issues in EC.

In summary, we demonstrate that genetic inhibition of AXL via shRNA is sufficient to significantly reduce EC metastatic progression and highlight clinical implications of AXL therapy for treatment of advanced or recurrent EC, against which current treatments can be significantly improved.

## MATERIALS AND METHODS

### Human Tissue Microarrays and Immunohistochemical Analysis

Commercial endometrial human tissue microarrays (TMAs) were obtained from US Biomax (Rockville, MD). Additionally, a tissue microarray containing sample cores from 10 patients with uterine papillary serous carcinoma who had undergone surgery at the University of Chicago between 1998 and 2007 was obtained from Ernst Lengyel (University of Chicago, IL) [58]. Another tissue microarray with corresponding survival data was constructed from patients treated at Washington University, under IRB approval. IHC was performed according to standard protocols. Briefly, slides were deparaffinized with xylene, rehydrated according to standard immunohistochemical methods, and stained with either anti-AXL primary antibody (R&D Systems, AF154; 1:200) or no primary (negative controls). Slides were stained using the VECTASTAIN ABC system (Vector Laboratories, Burlingame, CA) and DAB Substrate Kit for Peroxidase (Vector Laboratories) and counterstained with hematoxylin. For the US Biomax and U of Chicago TMAs, tumor cell expression of membranous AXL was scored semiquantitatively according to the percentage of cells positive for AXL expression (0 for 0%, 1 for 1-19%, 2 for 20-59%, and 3 for > 60%). For the Washington University TMA, AXL expression was recorded as a continuous variable. Data from this TMA was incorporated into Table 1 according to the same scoring system (0 for 0%, 1 for 1-19%, 2 for 20-59%, and 3 for > 60%). For the survival curve, IHC scores, 5–10% for low and 60–100% for high, was found to show the greatest survival difference between groups. Samples included various histologies including serous, mixed types, and endometrioid. Survival intervals were calculated from the date of surgery to death, or censored at the last date of visit.

### Cell lines

Endometrial ARK1 cells were provided by Shi-Wen Jiang (Mercer University School of Medicine, Savannah, GA, USA). Hec50a cells were a generous gift from Kimberly Leslie (University of Iowa Carver College of Medicine, Iowa City, IA, USA). AN3CA and KLE were obtained from American Type Culture Collection (Rockville, MD, USA), and Ishikawa cells were provided by Stuart Adler (Washington University School of Medicine, St. Louis, MO, USA). All cell lines were maintained in DMEM (Sigma, St. Louis, MO) supplemented with 10% heat inactivated FBS (Sigma, St. Louis, MO) and 1% penicillin and streptomycin (Invitrogen, Carlsbad, CA) at 37°C in a 5% CO_2_ incubator.

### shRNA constructs and lentivirus transduction

ARK1 and Hec50a were transduced with lentivirus carrying either oligos encoding an AXL shRNA or a scrambled sequence (shSCRM). shAXL oligos were synthesized as previously described 5′-GATTTGGAGAACACACTGA-3′ [37]. A scrambled sequence was used as a non-targeting shRNA control 5′-AATTGTACTACACAAAAGTAC-3′. Oligos were cloned into the pSiren RetroQ (BD Bioscience) vector and infected ARK1 and Hec50a cells, which were then selected in puromycin (Sigma). For *in vivo* luciferase studies, ARK1 cells were transduced with retrovirus particles encoding for the expression of firefly luciferase gene (FLuc).

### RNA interference

Human AXL siRNA (ON-TARGETplus SMART pool #L-003104-00-0005) and siControl (ON-TARGETplus Non-targeting pool #D-001810-10-05) were from Dharmacon (Lafayette, CO). One day prior to transfection, ARK1 cells were plated in a 60 mm dish at 50% confluence in DMEM medium supplemented with 10% FBS without antibiotics. Cells were transfected with 10 nM siRNAs using DharmaFECT 3 transfection reagent (T-2003-02) according to the manufacturer's instructions. Two days following transfection, ARK1 cells were plated into a 96-well plate for proliferation assays and harvested for western blot analysis of AXL expression at 24, 48, 72, and 96 hrs. Invasion and migration assays were also plated 48 hours following siRNA transfection.

### Proliferation assays

Cells were plated at densities of 4,000 cells per well into four 96-well plates in normal medium. Proliferation was measured at 24, 48, 72, and 96 hours using an 2,3-bis[2-methoxy-4-nitro-5-sulfophenyl]-2*H*-tetrazolium-5-carboxanilide inner salt (XTT)-based assay (Roche Molecular Biochemicals) as previously described [59]. Each experiment was done in triplicate and repeated three times.

### Transwell invasion and migration assays

Matrigel invasion assays were performed according to the manufacturer's protocol (Corning). Briefly, cells were serum starved for 24 hours and plated onto matrigel coated boyden chambers. Invasion assays were stained and analyzed after 16-48 hours. Migration assays were performed in a similar fashion, in uncoated Boyden chambers. Invaded cells were quantified by counting the number of invading cells per high-powered field. After 48 hours, invading cells on the bottom side of the membranes were fixed, stained, and counted in four different fields at 20X magnification. Migration of siRNA-treated ARK1 cells was counted at 10X magnification due to the distribution pattern of the migrated cells. The experiments were performed in triplicate wells and repeated at least three times.

For R428 invasion, ARK1 shSCRM and shAXL cells were serum-starved overnight and then treated with either serum-free media or 10 μM R428 (Selleckchem) for 4 hours before plating into Matrigel chambers. Invading cells were fixed and stained after 48 hours.

### Western blot analysis

Protein lysates were prepared from cultured cells or frozen tumors using a 9M Urea, 0.075 M Tris buffer (pH 7.6) and quantified using the Bradford assay. 50-100μg of protein was subjected to reducing SDS/PAGE by standard methods. Western blots were incubated with primary antibodies against AXL (R&D Systems), P-AXL (Cell Signaling), GAS-6 (R&D), AKT (Cell Signaling), and phospho-AKT (Cell Signaling) as needed. To confirm equal protein loading, blots were probed with antibodies specific for β-actin (Sigma Aldrich), HSP70 (Thermo Fisher), or Vinculin (BD Pharmingen).

### Zymogram

Hec50a shSCRM/AXL cells were starved in serum free media for 48 hours and 25,000 cells were plated into a 96 well plate in SF media. Conditioned media was collected 24 hours later. Equal volumes of conditioned media were run under non-reducing conditions on 10% gelatin zymogram gels (BioRad). After electrophoresis, gels were incubated in zymogram renature buffer (BioRad) for 30 min at RT and then incubated overnight in zymogram development buffer (BioRad) at 37°C. Gels were stained for 30 min with Coomassie Brilliant Blue R250 and destained in 40% methanol and 10% glacial acetic acid.

### RT-PCR

The RNeasy mini kit (Qiagen) was used to isolate RNA, 1 μg of which was used to make cDNA using the Quantitect Reverse Transcription kit (Qiagen) according to the manufacturer's protocol. RT-PCR was performed on 30 ng of cDNA using Fast SYBR Green Master Mix and the 7500 Fast Real-time PCR System (Applied Biosystems). Sequences of primers used were: 18S Fwd: 5-GCCCGAAGCGTTTACTTTGA-3 Rev: 5-TCCATTATTCCTAGCTGCGGTATC-3 [60]; AXL Fwd: 5-GTGGGCAACCCAGGGAATATC-3 Rev: 5-GTACTGTCCCGTGTCGGAAAG-3 [60]; MMP-1: Fwd: 5-ACACATCTGACCTACAGGATTGA-3 Rev: 5-GTGTGACATTACTCCAGAGTTGG-3 [24]; MMP-2 Fwd: 5-GCCCCAGACAGGTGATCTTG-3 Rev: 5-GCTTGCGAGGGAAGAAGTTGT-3 [24]; MMP-3 Fwd: 5-TTCCTGGCATCCCGAAGTGG-3 Rev: 5-ACAGCCTGGAGAATGTGAGTGG-3 [61]; MMP-9 FWD 5-GGGACGCAGACATCGTCATC-3 REV: 5-TCGTCATCGTCGAAATGGGC-3 [24]; uPA Fwd: 5-AGGGCAGCACTGTGAAATAGATAAGT-3 Rev: 5-CATGGTACGTTTGCTGAAGGA-3 [62]. Fold change in mRNA expression was calculated by the delta-delta CT method. Expression of corresponding genes was normalized to that of the housekeeping gene 18S. Each value was measured in triplicate, and the experiment was repeated at least twice.

### Orthotopic Model of Uterine Cancer

All procedures involving animals and their care were performed in accordance with the guidelines of the American Association for Accreditation for Laboratory Animal Care and the U.S. Public Health Service Policy on Humane Care and Use of Laboratory Animals. All animal studies were also approved and supervised by the Washington University Institutional Animal Care and Use Committee in accordance with the Animal Welfare Act, the Guide for the Care and Use of Laboratory Animals, and NIH guidelines.

ARK1 cells were transduced with retrovirus particles encoding firefly luciferase, then transfected with shSCRM and shAXL as described above. shSCRM and shAXL ARK1 cells (10×10^6^ cells in 0.5 ml of PBS) were injected intraperitoneally (i.p.) into female 6-to-8-week old (*n*=9) (NOD) SCID (Jackson Laboratory). Mice were monitored for adverse events until sacrifice. Approximately 35 days after injection, mice was subjected to bioluminescence imaging to monitor tumor growth as previously described [63]. Briefly, living mice were injected with 150 mg/mL D-luciferin (Gold Biotech) in PBS and imaged with a charge-coupled device (CCD) camera-based bioluminescence imaging system (IVIS50; Perkin-Elmer, Hopkinton, MA; exposure time 10s - 1minute, binning 8, field of view 12, f/stop 1, open filter). Bioluminescence signal was displayed as photons/sec/cm^2^/sr. At the completion of each experiment (60 days), mice were sacrificed, and aggregate tumor weight, location, and number of tumor nodules were recorded for each group. Tumor samples were fixed in formalin and embedded in paraffin for further analysis.

### Immunohistochemical analysis of Ki-67 expression in mouse xenografts

Mouse xenograft tumors were placed in fresh formalin for a minimum of 24 hours before processing for paraffin embedding. Sections were cut at 5 μm and mounted on glass slides. Slides were deparaffinized and hydrated in Xylene, gradually diluted in ethanol and water followed by antigen retrieval in Sodium Citrate at pH 6. Slides were probed with 1:2000 diluted Ki67 (AbCam) at 4C overnight, then washed and incubated with HRP conjugated secondary antibody. Antibody complexes were detected with 3,3′-diaminobenzidine and counter stained with Mayer's Hematoxylin.

### Statistical Analyses

Prism software (GraphPad) was used for statistical analyses. Two-tailed unpaired Student's *t* tests were performed to analyze statistical differences between groups. P values < 0.05 were considered statistically significant.

## SUPPLEMENTARY MATERIALS FIGURES



## References

[1] Siegel RL, Miller KD, Jemal A (2016). Cancer statistics, 2016. CA Cancer J Clin.

[2] Lewin SN, Herzog TJ, Barrena Medel NI, Deutsch I, Burke WM, Sun X, Wright JD (2010). Comparative performance of the 2009 international Federation of gynecology and obstetrics' staging system for uterine corpus cancer. Obstet Gynecol.

[3] Boruta DM, Gehrig PA, Fader AN, Olawaiye AB (2009). Management of women with uterine papillary serous cancer: a Society of Gynecologic Oncology (SGO) review. Gynecol Oncol.

[4] Moore KN, Fader AN (2011). Uterine papillary serous carcinoma. Clin Obstet Gynecol.

[5] Koh WJ, Greer BE, Abu-Rustum NR, Apte SM, Campos SM, Chan J, Cho KR, Cohn D, Crispens MA, Dupont N, Eifel PJ, Fader AN, Fisher CM (2014). Uterine neoplasms, version 1. 2014. J Natl Compr Canc Netw.

[6] McMeekin S, Dizon D, Barter J, Scambia G, Manzyuk L, Lisyanskaya A, Oaknin A, Ringuette S, Mukhopadhyay P, Rosenberg J, Vergote I (2015). Phase III randomized trial of second-line ixabepilone versus paclitaxel or doxorubicin in women with advanced endometrial cancer. Gynecol Oncol.

[7] Fleming GF (2007). Systemic chemotherapy for uterine carcinoma: metastatic and adjuvant. J Clin Oncol.

[8] Thigpen JT, Brady MF, Homesley HD, Malfetano J, DuBeshter B, Burger RA, Liao S (2004). Phase III trial of doxorubicin with or without cisplatin in advanced endometrial carcinoma: a gynecologic oncology group study. J Clin Oncol.

[9] Sasaki T, Knyazev PG, Clout NJ, Cheburkin Y, Gohring W, Ullrich A, Timpl R, Hohenester E (2006). Structural basis for Gas6-Axl signalling. The EMBO journal.

[10] Wu X, Liu X, Koul S, Lee CY, Zhang Z, Halmos B (2014). AXL kinase as a novel target for cancer therapy. Oncotarget.

[11] Bellosta P, Costa M, Lin DA, Basilico C (1995). The receptor tyrosine kinase ARK mediates cell aggregation by homophilic binding. Mol Cell Biol.

[12] Heide I, Sokoll AC, Henz BM, Nagel S, Kreissig K, Grutzkau A, Grabbe J, Wittig B, Neubauer A (1998). Regulation and possible function of axl expression in immature human mast cells. Ann Hematol.

[13] Braunger J, Schleithoff L, Schulz AS, Kessler H, Lammers R, Ullrich A, Bartram CR, Janssen JW (1997). Intracellular signaling of the Ufo/Axl receptor tyrosine kinase is mediated mainly by a multi-substrate docking-site. Oncogene.

[14] Linger RM, Keating AK, Earp HS, Graham DK (2008). TAM receptor tyrosine kinases: biologic functions, signaling, and potential therapeutic targeting in human cancer. Advances in cancer research.

[15] Nagata K, Ohashi K, Nakano T, Arita H, Zong C, Hanafusa H, Mizuno K (1996). Identification of the product of growth arrest-specific gene 6 as a common ligand for Axl, Sky, and Mer receptor tyrosine kinases. J Biol Chem.

[16] Demarchi F, Verardo R, Varnum B, Brancolini C, Schneider C (2001). Gas6 anti-apoptotic signaling requires NF-kappa B activation. J Biol Chem.

[17] Goruppi S, Ruaro E, Varnum B, Schneider C (1997). Requirement of phosphatidylinositol 3-kinase-dependent pathway and Src for Gas6-Axl mitogenic and survival activities in NIH 3T3 fibroblasts. Mol Cell Biol.

[18] Hafizi S, Dahlback B (2006). Signalling and functional diversity within the Axl subfamily of receptor tyrosine kinases. Cytokine Growth Factor Rev.

[19] Sharif MN, Sosic D, Rothlin CV, Kelly E, Lemke G, Olson EN, Ivashkiv LB (2006). Twist mediates suppression of inflammation by type I IFNs and Axl. The Journal of experimental medicine.

[20] Stenhoff J, Dahlback B, Hafizi S (2004). Vitamin K-dependent Gas6 activates ERK kinase and stimulates growth of cardiac fibroblasts. Biochem Biophys Res Commun.

[21] Yanagita M, Arai H, Nakano T, Ohashi K, Mizuno K, Fukatsu A, Doi T, Kita T (2001). Gas6 induces mesangial cell proliferation via latent transcription factor STAT3. J Biol Chem.

[22] Mishra A, Wang J, Shiozawa Y, McGee S, Kim J, Jung Y, Joseph J, Berry JE, Havens A, Pienta KJ, Taichman RS (2012). Hypoxia stabilizes GAS6/Axl signaling in metastatic prostate cancer. Molecular cancer research.

[23] Paccez JD, Vasques GJ, Correa RG, Vasconcellos JF, Duncan K, Gu X, Bhasin M, Libermann TA, Zerbini LF (2013). The receptor tyrosine kinase Axl is an essential regulator of prostate cancer proliferation and tumor growth and represents a new therapeutic target. Oncogene.

[24] Rankin EB, Fuh KC, Taylor TE, Krieg AJ, Musser M, Yuan J, Wei K, Kuo CJ, Longacre TA, Giaccia AJ (2010). AXL is an essential factor and therapeutic target for metastatic ovarian cancer. Cancer research.

[25] Shiozawa Y, Pedersen EA, Patel LR, Ziegler AM, Havens AM, Jung Y, Wang J, Zalucha S, Loberg RD, Pienta KJ, Taichman RS (2010). GAS6/AXL axis regulates prostate cancer invasion, proliferation, and survival in the bone marrow niche. Neoplasia.

[26] Sun W, Fujimoto J, Tamaya T (2004). Coexpression of Gas6/Axl in human ovarian cancers. Oncology.

[27] Sun WS, Fujimoto J, Tamaya T (2003). Coexpression of growth arrest-specific gene 6 and receptor tyrosine kinases Axl and Sky in human uterine endometrial cancers. Ann Oncol.

[28] Wang X, Saso H, Iwamoto T, Xia W, Gong Y, Pusztai L, Woodward WA, Reuben JM, Warner SL, Bearss DJ, Hortobagyi GN, Hung MC, Ueno NT (2013). TIG1 promotes the development and progression of inflammatory breast cancer through activation of Axl kinase. Cancer Res.

[29] Zhang YX, Knyazev PG, Cheburkin YV, Sharma K, Knyazev YP, Orfi L, Szabadkai I, Daub H, Keri G, Ullrich A (2008). AXL is a potential target for therapeutic intervention in breast cancer progression. Cancer Res.

[30] Shieh YS, Lai CY, Kao YR, Shiah SG, Chu YW, Lee HS, Wu CW (2005). Expression of axl in lung adenocarcinoma and correlation with tumor progression. Neoplasia.

[31] Wu F, Li J, Jang C, Wang J, Xiong J (2014). The role of Axl in drug resistance and epithelial-to-mesenchymal transition of non-small cell lung carcinoma. Int J Clin Exp Pathol.

[32] Asiedu MK, Beauchamp-Perez FD, Ingle JN, Behrens MD, Radisky DC, Knutson KL (2014). AXL induces epithelial-to-mesenchymal transition and regulates the function of breast cancer stem cells. Oncogene.

[33] Gjerdrum C, Tiron C, Hoiby T, Stefansson I, Haugen H, Sandal T, Collett K, Li S, McCormack E, Gjertsen BT, Micklem DR, Akslen LA, Glackin C, Lorens JB (2010). Axl is an essential epithelial-to-mesenchymal transition-induced regulator of breast cancer metastasis and patient survival. Proc Natl Acad Sci U S A.

[34] Holland SJ, Pan A, Franci C, Hu Y, Chang B, Li W, Duan M, Torneros A, Yu J, Heckrodt TJ, Zhang J, Ding P, Apatira A (2010). R428, a selective small molecule inhibitor of Axl kinase, blocks tumor spread and prolongs survival in models of metastatic breast cancer. Cancer research.

[35] Li Y, Ye X, Tan C, Hongo JA, Zha J, Liu J, Kallop D, Ludlam MJ, Pei L (2009). Axl as a potential therapeutic target in cancer: role of Axl in tumor growth, metastasis and angiogenesis. Oncogene.

[36] Vajkoczy P, Knyazev P, Kunkel A, Capelle HH, Behrndt S, von Tengg-Kobligk H, Kiessling F, Eichelsbacher U, Essig M, Read TA, Erber R, Ullrich A (2006). Dominant-negative inhibition of the Axl receptor tyrosine kinase suppresses brain tumor cell growth and invasion and prolongs survival. National Academy of Sciences of the United States of America.

[37] Holland SJ, Powell MJ, Franci C, Chan EW, Friera AM, Atchison RE, McLaughlin J, Swift SE, Pali ES, Yam G, Wong S, Lasaga J, Shen MR (2005). Multiple roles for the receptor tyrosine kinase axl in tumor formation. Cancer Res.

[38] Egeblad M, Werb Z (2002). New functions for the matrix metalloproteinases in cancer progression. Nat Rev Cancer.

[39] Kessenbrock K, Plaks V, Werb Z (2010). Matrix metalloproteinases: regulators of the tumor microenvironment. Cell.

[40] Liotta LA, Tryggvason K, Garbisa S, Hart I, Foltz CM, Shafie S (1980). Metastatic potential correlates with enzymatic degradation of basement membrane collagen. Nature.

[41] Dery MC, Chaudhry P, Leblanc V, Parent S, Fortier AM, Asselin E (2011). Oxytocin increases invasive properties of endometrial cancer cells through phosphatidylinositol 3-kinase/AKT-dependent up-regulation of cyclooxygenase-1, -2, and X-linked inhibitor of apoptosis protein. Biol Reprod.

[42] Lin CY, Chao A, Wang TH, Hsueh S, Lee YS, Wu TI, Chao AS, Huang HJ, Chou HH, Chang TC, Lai CH (2014). A dual tyrosine kinase inhibitor lapatinib suppresses overexpression of matrix metallopeptidase 1 (MMP1) in endometrial cancer. J Mol Med (Berl).

[43] Mannelqvist M, Stefansson IM, Bredholt G, Hellem Bo T, Oyan AM, Jonassen I, Kalland KH, Salvesen HB, Akslen LA (2011). Gene expression patterns related to vascular invasion and aggressive features in endometrial cancer. Am J Pathol.

[44] Xu H, Chen X, Huang J, Deng W, Zhong Q, Yue C, Wang P, Huang Z (2013). Identification of GPR65, a novel regulator of matrix metalloproteinases using high through-put screening. Biochem Biophys Res Commun.

[45] Huang CY, Chang MC, Huang WY, Huang CT, Tang YC, Huang HD, Kuo KT, Chen CA, Cheng WF (2015). Urokinase-type plasminogen activator resulting from endometrial carcinogenesis enhances tumor invasion and correlates with poor outcome of endometrial carcinoma patients. Sci Rep.

[46] Yoneda J, Kuniyasu H, Crispens MA, Price JE, Bucana CD, Fidler IJ (1998). Expression of angiogenesis-related genes and progression of human ovarian carcinomas in nude mice. J Natl Cancer Inst.

[47] Nogami Y, Banno K, Kisu I, Yanokura M, Umene K, Masuda K, Kobayashi Y, Yamagami W, Nomura H, Tominaga E, Susumu N, Aoki D (2013). Current status of molecular-targeted drugs for endometrial cancer (Review). Mol Clin Oncol.

[48] Silva JL, Paulino E, Dias MF, Melo AC (2015). Endometrial cancer: redefining the molecular-targeted approach. Cancer Chemother Pharmacol.

[49] Weber GF (2013). Why does cancer therapy lack effective anti-metastasis drugs?. Cancer letters.

[50] Tecimer C, Doering DL, Goldsmith LJ, Meyer JS, Abdulhay G, Wittliff JL (2001). Clinical relevance of urokinase-type plasminogen activator, its receptor, and its inhibitor type 1 in endometrial cancer. Gynecol Oncol.

[51] Lee HJ, Jeng YM, Chen YL, Chung L, Yuan RH (2014). Gas6/Axl pathway promotes tumor invasion through the transcriptional activation of Slug in hepatocellular carcinoma. Carcinogenesis.

[52] Byers LA, Diao L, Wang J, Saintigny P, Girard L, Peyton M, Shen L, Fan Y, Giri U, Tumula PK, Nilsson MB, Gudikote J, Tran H, Cardnell RJ, Bearss DJ, Warner SL (2013). An epithelial-mesenchymal transition gene signature predicts resistance to EGFR and PI3K inhibitors and identifies Axl as a therapeutic target for overcoming EGFR inhibitor resistance. Clinical cancer research.

[53] Liu L, Greger J, Shi H, Liu Y, Greshock J, Annan R, Halsey W, Sathe GM, Martin AM, Gilmer TM (2009). Novel mechanism of lapatinib resistance in HER2-positive breast tumor cells: activation of AXL. Cancer Res.

[54] Sequist LV, Waltman BA, Dias-Santagata D, Digumarthy S, Turke AB, Fidias P, Bergethon K, Shaw AT, Gettinger S, Cosper AK, Akhavanfard S, Heist RS, Temel J (2011). Genotypic and histological evolution of lung cancers acquiring resistance to EGFR inhibitors. Science translational medicine.

[55] Vuoriluoto K, Haugen H, Kiviluoto S, Mpindi JP, Nevo J, Gjerdrum C, Tiron C, Lorens JB, Ivaska J (2011). Vimentin regulates EMT induction by Slug and oncogenic H-Ras and migration by governing Axl expression in breast cancer. Oncogene.

[56] Ware KE, Hinz TK, Kleczko E, Singleton KR, Marek LA, Helfrich BA, Cummings CT, Graham DK, Astling D, Tan AC, Heasley LE (2013). A mechanism of resistance to gefitinib mediated by cellular reprogramming and the acquisition of an FGF2-FGFR1 autocrine growth loop. Oncogenesis.

[57] Zhang Z, Lee JC, Lin L, Olivas V, Au V, LaFramboise T, Abdel-Rahman M, Wang X, Levine AD, Rho JK, Choi YJ, Choi CM, Kim SW (2012). Activation of the AXL kinase causes resistance to EGFR-targeted therapy in lung cancer. Nature genetics.

[58] Tergas AI, Buell-Gutbrod R, Gwin K, Kocherginsky M, Temkin SM, Fefferman A, Lengyel E, Yamada SD (2012). Clinico-pathologic comparison of type II endometrial cancers based on tamoxifen exposure. Gynecol Oncol.

[59] Heinrich MC, Griffith DJ, Druker BJ, Wait CL, Ott KA, Zigler AJ (2000). Inhibition of c-kit receptor tyrosine kinase activity by STI 571, a selective tyrosine kinase inhibitor. Blood.

[60] Rankin EB, Fuh KC, Castellini L, Viswanathan K, Finger EC, Diep AN, LaGory EL, Kariolis MS, Chan A, Lindgren D, Axelson H, Miao YR, Krieg AJ, Giaccia AJ (2014). Direct regulation of GAS6/AXL signaling by HIF promotes renal metastasis through SRC and MET. Proc Natl Acad Sci U S A.

[61] Taddei ML, Cavallini L, Comito G, Giannoni E, Folini M, Marini A, Gandellini P, Morandi A, Pintus G, Raspollini MR, Zaffaroni N, Chiarugi P (2014). Senescent stroma promotes prostate cancer progression: the role of miR-210. Mol Oncol.

[62] Majidzadeh AK, Esmaeili R, Abdoli N (2011). TFRC and ACTB as the best reference genes to quantify Urokinase Plasminogen Activator in breast cancer. BMC Res Notes.

[63] Gross S, Piwnica-Worms D (2005). Real-time imaging of ligand-induced IKK activation in intact cells and in living mice. Nat Methods.

