# Supplementary material for: AXL modulates extracellular matrix protein expression and is essential for invasion and metastasis in endometrial cancer
Source: Oncotarget. 2016 Oct 13;7(47):77291–305. doi: 10.18632/oncotarget.12637 (PMC5340229; doi:10.18632/oncotarget.12637)
Supplement: Supplementary file 1 [file oncotarget-07-77291-s001.pdf]

AXL modulates extracellular matrix protein expression and is essential for invasion and metastasis in endometrial cancer

SUPPLEMENTARY FIGURES

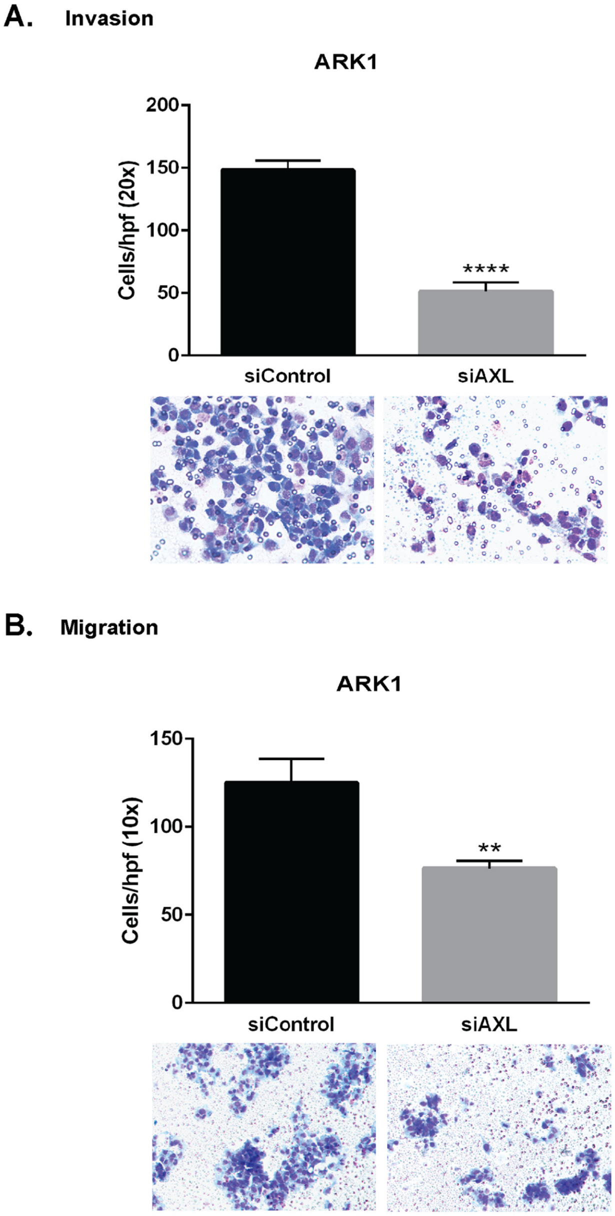

Supplementary Figure S1: Additional genetic knockdown of AXL confirms the role of AXL in invasion and migration of endometrial cancer cells. A. Matrigel invasion assay of ARK1 siControl and siAXL cells plated 48hrs following siRNA transfection. Images were taken at 48h at 20X magnification showing fewer invading cells for siAXL cells. Graphs show quantification of invasion assays (n=3). Data are represented as mean +/- SEM and asterisks indicate a significant increase or decrease in expression compared with shSCRM as determined by student's t test (****, P<0.0001). **B.** Migration assay using boyden chambers of ARK1 siControl and siAXL cells plated 48hrs following siRNA transfection. Images were taken after 48h at 10X magnification illustrating inhibition of siAXL cells' migration. Graphs show quantification of migration assays (n=3). Data are represented as mean +/- SEM and asterisks indicating statistical significance compared to shSCRM as determined by the student's t-test (**, P < 0.01).

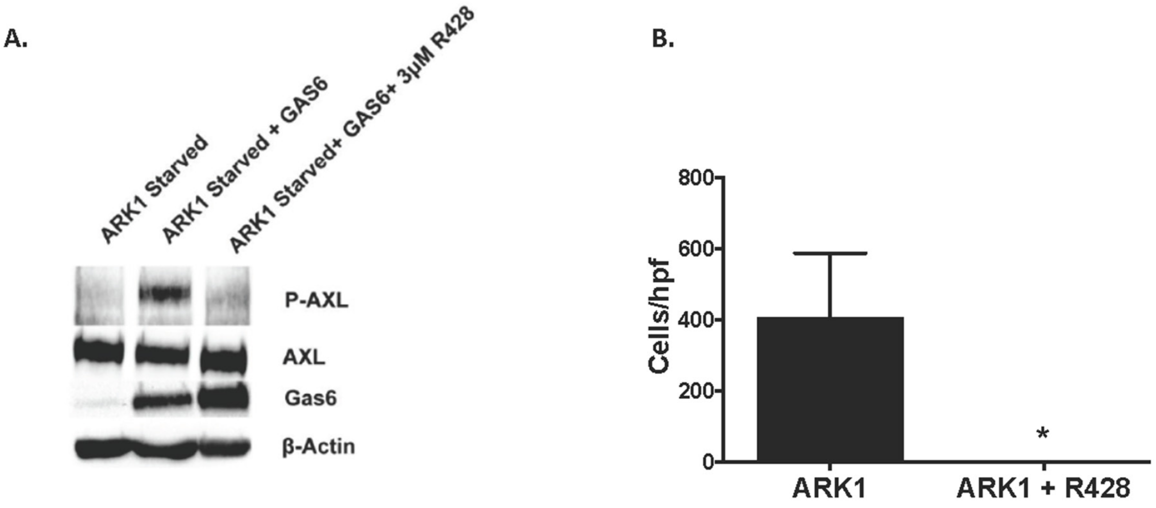

Supplementary Figure S2: R428 inhibits P-AXL expression and cellular invasion. A. Western blot analysis of AXL (140 kDa), phospho-AXL (Tyr702, 140 kDa), and GAS6 (75 kDa) expression in ARK1 cells. Briefly, ARK1 parental cells were serum-starved for 48h, followed by 4hours of 3 µM R428 treatment and then 15mins of 600ng/ml GAS6 stimulation. β-actin (42 kDa) is shown as a loading control. **B.** Matrigel invasion assay of ARK1 cells with and without R428. Cells were starved overnight and then either treated with serum-free media or 10 µM R428 for 4 hours. Images were taken at 48h at 20X magnification showing fewer invading cells for R428 treated cell. Graphs show quantification of one invasion assay done in triplicate. Data are represented as mean +/- SEM and asterisks indicate a significant increase or decrease in expression compared with its control group as determined by student's t test (*, $P < 0.01$).

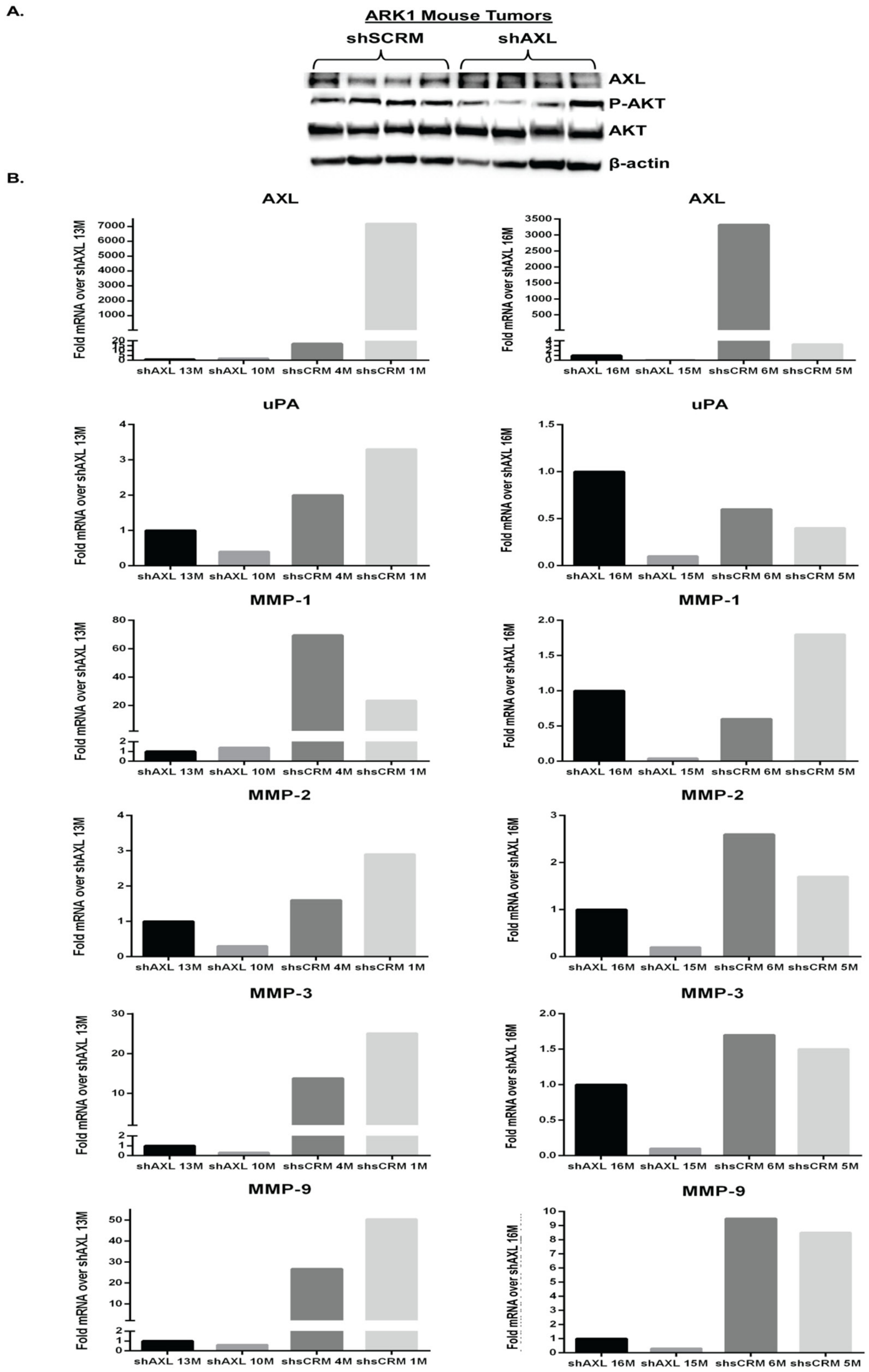

Supplementary Figure S3: ARK1 shSCRM and shAXL mouse tumors confirm in vitro AXL knockdown and decreased MMP expression. A. Western blot analysis of AXL (140 kDa), phospho-AKT (Ser 473, 56-60 kDa), and total AKT (60 kDa) expression in ARK1 mouse tumor cells expressing shSCRM or shAXL. β-actin (42 kDa) is shown as a loading control. **B.** Real time PCR analysis of MMP-1,-2,-3,-9, uPA, and AXL expression in ARK1 shAXL and shSCRM mouse tumors. All expression values were normalized to 18S and fold changes were calculated over one designated shSCRM.

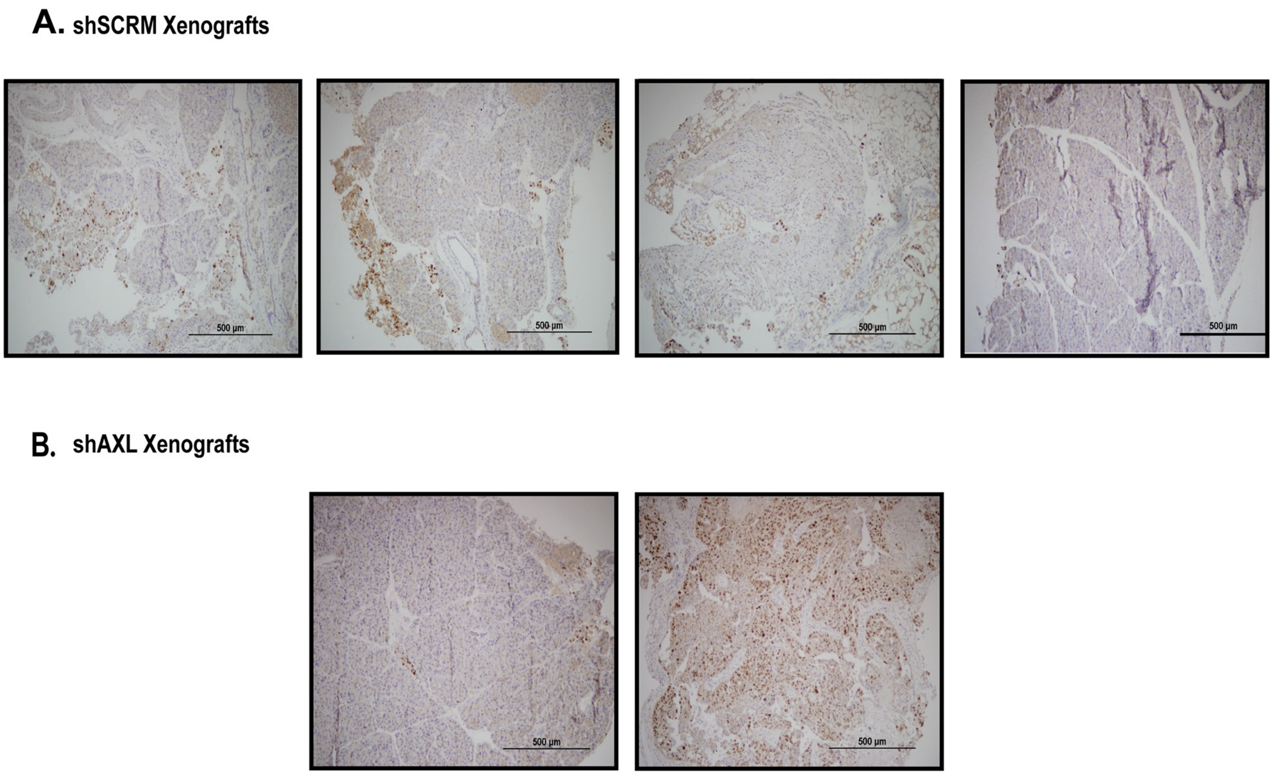

Supplementary Figure S4: Ki-67 staining of mouse xenograft tumors indicates no difference in proliferation. A. Representative images of Immunohistochemical analysis of Ki-67 expression in mouse xenografts generated from shSCRM cells; Scale bars: 500 µm. **B.** Representative images of Immunohistochemical analysis of Ki-67 expression in xenografts generated from shAXL cells; Scale bars: 500 µm..
